# A 3-year follow-up study of atropine treatment for progressive myopia in Europeans

**DOI:** 10.1038/s41433-020-1122-7

**Published:** 2020-09-21

**Authors:** Jan Roelof Polling, Emily Tan, Sjoerd Driessen, Sjoukje E. Loudon, Hoi-Lam Wong, Astrid van der Schans, J. Willem L. Tideman, Caroline C. W. Klaver

**Affiliations:** 1 Department Ophthalmology, Erasmus Medical Center, Rotterdam, The Netherlands; 2 Department Optometry & Orthoptics, Faculty of Health, University of Applied Sciences, Utrecht, The Netherlands; 3 Department Epidemiology, Erasmus Medical Center, Rotterdam, The Netherlands; 4 Department of Ophthalmology, Radboud University Medical Centre, Nijmegen, Gelderland The Netherlands; 5 Institute of Molecular and Clinical Ophthalmology, Basel, Switzerland

**Keywords:** Outcomes research, Drug therapy

## Abstract

**Background:**

Atropine is the most powerful treatment for progressive myopia in childhood. This study explores the 3-year effectiveness of atropine in a clinical setting.

**Methods:**

In this prospective clinical effectiveness study, children with progressive myopia ≥ 1D/year or myopia ≤ −2.5D were prescribed atropine 0.5%. Examination, including cycloplegic refraction and axial length (AL), was performed at baseline, and follow-up. Outcome measures were spherical equivalent (SER) and AL; annual progression of SER on treatment was compared with that prior to treatment. Adjustments to the dose were made after 1 year in case of low (AL ≥ 0.3 mm/year) or high response (AL < 0.1 mm/year) of AL.

**Results:**

A total of 124 patients were enrolled in the study (median age: 9.5, range: 5–16 years). At baseline, median SER was −5.03D (interquartile range (IQR): 3.08); median AL was 25.14 mm (IQR: 1.30). *N* = 89 (71.8%) children were persistent to therapy throughout the 3-year follow-up. Median annual progression of SER for these children was −0.25D (IQR: 0.44); of AL 0.11 mm (IQR: 0.18). Of these, *N* = 32 (36.0%) had insufficient response and were assigned to atropine 1%; *N* = 26 (29.2%) showed good response and underwent tapering in dose. Rebound of AL progression was not observed. Of the children who ceased therapy, *N* = 9 were lost to follow-up; *N* = 9 developed an allergic reaction; and *N* = 17 (19.1%) stopped due to adverse events.

**Conclusion:**

In children with or at risk of developing high myopia, a starting dose of atropine 0.5% was associated with decreased progression in European children during a 3-year treatment regimen. Our study supports high-dose atropine as a treatment option for children at risk of developing high myopia in adulthood.

## Abstract 2

**Learning Objectives:**

Upon completion of this activity, participants will be able to:

1. Distinguish the most salient pathological feature of high myopia.
2. Evaluate the long-term tolerability of atropine eye drops for myopia.
3. Analyze the long-term efficacy of atropine eye drops for myopia.
4. Assess variables that might alter the efficacy of atropine eye drops for myopia.

**Continuing Medical Education:**

In support of improving patient care, this activity has been planned and implemented by Medscape, LLC and Springer Nature. Medscape, LLC is jointly accredited by the Accreditation Council for Continuing Medical Education (ACCME), the Accreditation Council for Pharmacy Education (ACPE), and the American Nurses Credentialing Center (ANCC), to provide continuing education for the healthcare team.

Medscape, LLC designates this Journal-based CME activity for a maximum of 1.00 AMA PRA Category 1 Credit(s). Physicians should claim only the credit commensurate with the extent of their participation in the activity.

All other clinicians completing this activity will be issued a certificate of participation. To participate in this journal CME activity: (1) review the learning objectives and author disclosures; (2) study the education content; (3) take the post-test with a 75% minimum passing score and complete the evaluation at www.medscape.org/journal/eye; (4) view/print certificate.

**Credit hours:**

1.0

**Release date:**

**Expiration date: 21 September 2021**

**Post-test link:** https://medscape.org/eye/posttest934752
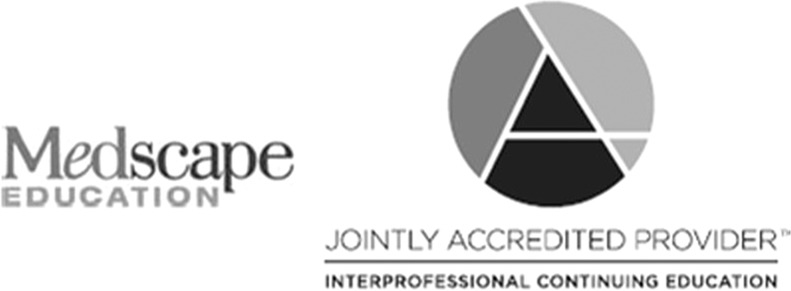

**Authors/Editors disclosure information:**

Sobha Sivaprasad, MD, has disclosed the following relevant financial relationships: served as an advisor or consultant for: Allergan, Inc.; Apellis; Bayer AG; Boehringer Ingelheim Pharmaceuticals, Inc.; Heidelberg Pharma GmbH; Novartis; Oculis; Optos; Oxurion; Roche. Served as a speaker or a member of a speakers bureau for: Allergan, Inc.; Bayer AG; Novartis Pharmaceuticals Corporation; Optos. Received grants for clinical research from: Allergan, Inc.; Bayer AG; Boehringer Ingelheim Pharmaceuticals, Inc.; Novartis Pharmaceuticals Corporation; Optos. Jan Roelof Polling, MD, has disclosed the following relevant financial relationships: served as an advisor or consultant for: Théa Laboratories; Nevakar, Inc. Emily Tan, MD, has disclosed no relevant financial relationships. Sjoerd Driessen, MD, has disclosed no relevant financial relationships. Sjoukje E. Loudon, MD, has disclosed no relevant financial relationships. Hoi-Lam Wong, MD, has disclosed no relevant financial relationships. Astrid van der Schans, MD, PhD, has disclosed no relevant financial relationships. J. Willem Tideman, MD, has disclosed no relevant financial relationships. Caroline C. W. Klaver, MD, PhD, has disclosed the following relevant financial relationships: served as an advisor or consultant for: Bayer AG; Nevakar, Inc.; Novartis Pharmaceuticals Corporation; Théa Laboratories.

**Journal CME author disclosure information:**

Charles P. Vega, MD, has disclosed the following relevant financial relationships: served as an advisor or consultant for: GlaxoSmithKline; Johnson & Johnson Pharmaceutical Research & Development, L.L.C. Served as a speaker or a member of a speakers bureau for: Genentech, Inc.; GlaxoSmithKline.

## Introduction

The prevalence of myopia is increasing all over the world, and has reached the highest frequencies in young adults in South Korea (96.5%), but has also increased significantly in Europe (49.2%) [1, 2]. The trait is determined by several optical components, of which increased axial length (AL) is the most important [3]. High myopia, i.e. refractive errors −6D or more, has increased from 4.2 to 21.6% in East-Asians and from 1.4 to 5.3% in Europeans [2, 4]. Countries which presently have a low prevalence will follow these trends, as myopia prevalence is driven by lifestyle changes such as less time outdoors and increased near work activities [5]. Myopia carries a significant risk of retinal detachment, glaucoma, and myopic macular degeneration, which is most prominent for severe refractive errors [6]. Of those with high myopia, one in three develops bilateral severe visual impairment or blindness with age [7]. This highlights the need for myopia control strategies in children with progressive myopia, in particular progression to high myopia [5, 8, 9].

During the last 10 years, many intervention studies for myopia progression have emerged [10–12]. Although lifestyle adjustments and optical solutions can be effective, pharmacological interventions targeting muscarinic receptors have shown the highest efficacy on reduction of eye growth [13, 14]. Atropine is a nonselective muscarinic receptor antagonist which has been tested for progressive myopia in several dosages [10]. High dosages, 0.5 and 1%, are the most effective in reducing eye growth, but have drawbacks as pupil dilatation, loss of accommodation, and potential rebound of spherical equivalent of refraction (SER) after stopping [15]. The lowest dose of atropine, 0.01%, has become popular because it has minimal side effects and virtually no rebound after stopping, but reduction on AL progression is also minimal [16–18].

In an earlier study, we reported 1 year results of intervention with atropine 0.5% for progressive myopia in a clinical setting in Europe. In children with already severe myopic refractive errors (mean SER, −6.6D) and progression of myopia 1D/year or more, we showed that atropine 0.5% reduced myopia progression to 0.1D/year. Despite the side effects, persistence to therapy was 78% [19]. We extended this study, and now report 3-year follow-up after the starting dose of atropine 0.5%. We addressed the photophobia and accommodation problems by prescribing photochromic multifocal spectacles.

## Materials and methods

### Study design and population

The design was a prospective clinic-based effectiveness study. The setting was a single center study in the Erasmus Medical Center in Rotterdam, the Netherlands, which included the Sophia Children’s hospital. Erasmus Medical Center has been a referral center for myopia control since 2010. Two examiners (JRP and AS) obtained cycloplegic refractive error and AL in the children throughout the study. Inclusion criteria have been described previously [19]. In short, consecutive children 5–16 years presenting with SER progression rate of at least 1D/year, or an SER of at least −2.5D in children 10 years and younger, or SER −5.0D in children aged 11 years or older were eligible. Exclusion criteria included those with pediatric pathology (e.g., amblyopia, strabismus, or systemic disorders) and low vision due to retinal dystrophies. The current report included children who presented at our clinic between March 2011 and January 2015. Children and parents received a patient information leaflet followed by oral consultation, and participants provided written informed parental consent (parents or legal guardians and children when age 12+ years; only parents and legal guardians when age < 12 years). All patients were scheduled for follow-up visits every 6 months from baseline onwards. The occurrence of serious adverse events was noted in the medical chart, and affected patients were referred to a specialist. The study adhered to the tenets of the Declaration of Helsinki, and was approved by the Institutional Review Board of the Erasmus Medical Center.

### Intervention

The intervention at baseline was atropine eye drops 0.5%; both eyes were treated before bedtime. After at least 1 year of atropine 0.5%, adjustments to the dose were made in case of insufficient response or stability of SER and AL. Insufficient response was considered present when myopia progressed ≥ −1D/year, and AL increased ≥ 0.3 mm/yr. Moderate response was defined as SER ≥ −0.5 to −1D/year and AL ≥ 0.2–0.3 mm/year; and good response as SER < −0.5D/year and AL < 0.2 mm/year [15]. In children with good response, atropine concentration was tapered to 0.25%, and further to 0.1 and 0.01% every 6 months when myopia progression remained stable. Increase of atropine concentration was indicated if the progression was moderate to insufficient. All dosages were distributed in multi dose bottles preserved with benzalkonium chloride, sodium edetate, boric acid, and purified water (FNA Dutch pharmacists).

### Eye examination

A standardized ophthalmological examination was performed at baseline, and at 6, 18, 24, 30, and 36 months. Baseline and follow-up measurements included a cycloplegic refractive error measurement with two drops of cyclopentolate 1% with 5 min interval and a minimum waiting time of 45 min after the first drop. In very dark irises with pupil diameter < 6 mm an additional drop of cyclopentolate was adjusted. In case of atropine 0.5 and 1% interventions, cycloplegia was considered already present. Refractive error was measured by using a Topcon auto refractor (KR8900). At least three measurements per eye were averaged to the mean refractive error per eye. SER was calculated as the average sphere + 1/2 cylinder of both eyes. AL was measured with the IOL Master (Carl Zeiss MEDITEC IOLMaster 500, Jena, Germany) and for AL five measurements per eye were averaged to a mean AL. The average AL of both eyes was used for the analysis. Best-corrected Snellen visual acuity was performed at 6 m distance with a decimal equivalent. The LogMAR based Dutch Radner chart was used to assess binocular reading visual acuity at 25 or 40 cm. To assess compliance with atropine eye drops, dynamic retinoscopy was performed according standard protocol to detect presence of accommodation paralysis and the Richmond Products Clear Pupilometer was used to measure pupil size (Albuquerque, NM, USA).

### Statistical analysis

Primary outcome was the annual progression rate of SER and AL for years 1–3. The pretreatment progression rate of SER was calculated using cycloplegic refractive error measurements obtained from medical records. Both SER and AL showed a skewed distribution; therefore medians were calculated as well as the interquartile range (IQR). Differences in outcomes between the various dosing regimens and between prolongation and cessation of therapy were assessed with Mann–Whitney *U* nonparametric test for continuous outcome measures, and with Fisher’s exact test for categorical outcome measures. Differences in progression rates in SER and AL were obtained with Wilcoxon signed-rank test. Correlation between annual progression of SER and AL was calculated with Pearson’s regression analysis. Throughout the study, *p* < 0.05 was used as criterion of statistical significance. All statistical tests were performed by using IBM SPSS Statistics for Windows, Version 24.0 (IBM Corp., Armonk, NY, USA).

## Results

The current analysis included 124 children who started atropine 0.5% treatment for progressive myopia. Informed consent was obtained from all parents of children and all children aged 12 years or older.

Demographics of the study population are summarized in Table 1. Gender was evenly distributed and the median age was 9.5 years (IQR: 4). The majority of children (66.9%) had European ethnicity. Median SER 1 year prior to the study was −3.88D (IQR: 4.00). At baseline, median SER was −5.03D (IQR: 3.08) demonstrating an annual progression rate of SER of more than 1D prior to treatment. High myopia (SER ≤ −6D) was present in 46 (37.1%) of children (range: −6.13 to −17.06D); median AL was 25.14 (IQR: 1.30). Parental myopia was reported by 80.6%; high parental myopia by 37.9%.

**Table 1:** Distribution of demographics and clinical measures of children eligible for the study.

Characteristics at baseline	
Patients, *N*	124
Gender, *N* (%)	
Female	67/124 (54%)
Median age in years (IQR)	9.5 (4)
Ethnicity^a^	
European	83/124 (66.9%)
East Asian	13/124 (10.5%)
Other^b^	29/124 (22.6%)
Parental presence of myopia, *N* (%)	
No myopia	12/124 (9.7%)
One parent	51/124 (41.1%)
Both parents	49/124 (39.5%)
Missing^c^	12/124 (9.7%)
Parental presence of high myopia^d^ (≤−6D), *N* (%)	47/124 (37.9%)
Median onset of myopia in years^e^ (IQR)	6 (3)
Median SE in D (IQR)	−5.03 (IQR: 3.08)
Median AL in mm (IQR)	25.14 (IQR: 1.30)

^a^Obtained by medical record.

^b^Other ethnicities included children with a background form Surinam, Venezuela, the Dutch Antilles, Indonesia, and Pakistan.

^c^Complete data could not be obtained due to adoption or one parent situation.

^d^In either parent or both parents obtained by questionnaire.

^e^Obtained by questionnaire.

Results of outcome and adherence are shown in Table 2. Of the 124 children, 89 (71.8%) stayed on treatment during the full 3 years of follow-up, of these, 31 (34.8%) stayed on 0.5% atropine, 32 (36.0%) increased in dose to 1%, and 26 (29.2%) children decreased in dose. Decreasing the dose did not lead to rebound growth of AL. Of those who ceased therapy, 9 (6.8%) children stopped due to an allergic reaction following the eye drops; 17 (13.6%) children stopped due to photophobia and non-eye-related adverse events; and 9 (6.8%) were lost to follow-up. The 17 children who ceased therapy due to adverse events did so primarily during the first 3 months of treatment. Risk factors for nonadherence were not significant although children who ceased therapy were somewhat older.

**Table 2:** Progression of spherical equivalent and axial length for children receiving atropine 0.5% as a starting dose.

	Continued therapy *N* = 89 (71.8%)			Ceased therapy *N* = 35 (28.2%)		
	Increased dose *N* = 32	Decreased dose *N* = 26	Same dose *N* = 31	Allergy stop^b^ *N* = 9	Adverse events^c^ *N* = 17	Lost to follow-up *N* = 9
Median age (year) myopia onset (IQR)	6.0 (3)	7.0 (4)	6.0 (4)	6.0 (5)	6.0 (5)	7.0 (6)
Median age (year) at baseline (IQR)	8.5 (3)	11.0 (4)	9.0 (3)	9.0 (4)	11.0 (5)	12.0 (6)
Median spherical equivalent (SE) in D						
1 year prior to treatment	−4.5 (4.9)	−2.9 (3.9)	−3.8 (3.1)	−3.6 (6.4)	−4.3 (4.5)	−4.8 (4.1)
Baseline	−5.8 (3.5)	−4.4 (2.8)	−4.9 (2.8)	−5.4 (4.9)	−5.3 (4.0)	−5.4 (3.0)
1st year	−6.0 (3.6)	−4.2 (3.5)	−4.8 (2.5)	−7.5 (6.7)	−5.6 (3.7)	–
2nd year	−6.9 (4.7)	−4.6 (2.8)	−5.2 (2.6)	−8.0 (5.5)	−6.8 (3.3)	–
3rd year	−7.5 (5.2)	−4.8 (2.6)	−5.6 (2.6)	−8.1 (6.0)	−7.8 (3.7)	–
Median progression rate of SE in D/year						
1 year before treatment	−1.0 (1.3)	−1.3 (1.0)	−1.0 (1.2)	−1.1 (2.1)	−0.8 (1.1)	−0.4 (1.0)
1st year	−0.4 (0.6)	+0.2 (0.7)	+0.1 (0.5)	−0.4 (0.7)	−0.7 (1.1)	–
2nd year	−0.6 (0.7)	−0.3 (0.4)	−0.3 (0.6)	−0.9 (1.3)	−0.8 (0.9)	–
3rd year	−0.5 (0.8)	−0.3 (0.3)	−0.3 (0.5)	−0.4 (1.4)	−0.9 (1.1)	–
Median axial length (AL) in mm^a^						
Baseline	25.2 (1.3)	24.7 (1.3)	25.4 (1.6)	25.2 (2.8)	24.8 (1.2)	25.9 (2.5)
1st year	25.5 (1.7)	24.5 (1.5)	25.3 (1.6)	25.4 (1.5)	25.1 (1.3)	–
2nd year	25.8 (1.4)	24.7 (1.3)	25.3 (1.6)	–	–	–
3rd year	25.9 (2.3)	24.8 (1.5)	25.4 (1.5)	–	–	–
Median progression rate AL in mm/year^a^						
1st year	0.3 (0.2)	0.0 (0.2)	0.0 (0.1)	0.2 (0.3)	0.3 (1.0)	–
2nd year	0.3 (0.3)	0.1 (0.1)	0.1 (0.2)	–	–	–
3rd year	0.2 (0.3)	0.1 (0.1)	0.1 (0.1)	–	–	–

^a^AL was not included in the standard ophthalmological examination 1 year prior to start of therapy and was not included in the children who stopped atropine treatment.

^b^Allergies developed after 1 year. First-year data are on treatment, 2nd and 3rd year were without treatment.

^c^Adverse events included photophobia, reading difficulties, nightmares, and deterioration of behavioral problems in a child with diagnosis of ADHD.

**Fig. 1:**
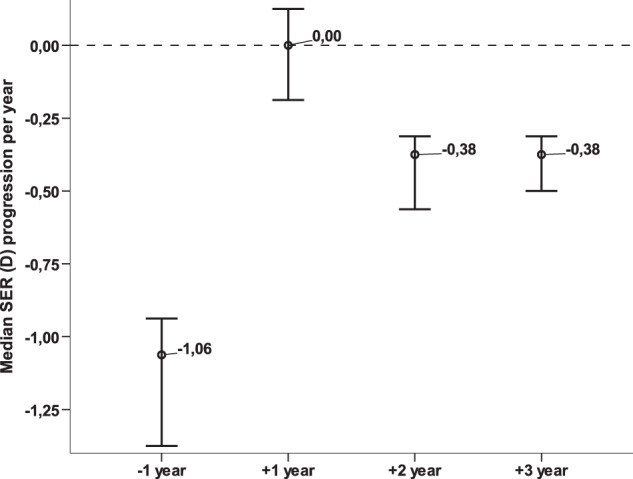
Median Spherical Equivalent (SER) change in dioptres per year in children treated with atropine 0.5% for progressive myopia. Error bars represent the 95% Confidence Interval.

In those who fulfilled 3 years of treatment, the median annual progression of SER was −0.25D (IQR: 0.44); of AL 0.11 mm (IQR: 0.18). Figure 1 represents the median annual progression rate of SER. Median progression was reduced to 0.00D in the 1st year, and −0.41 and −0.38D in the 2nd and 3rd year (all *p* < 0.01). Comparing these progressions to those prior to treatment, annual reduction rates of SER were 100, 65, and 68.2% (all *p* < 0.01; Fig. 1).

**Fig. 2:**
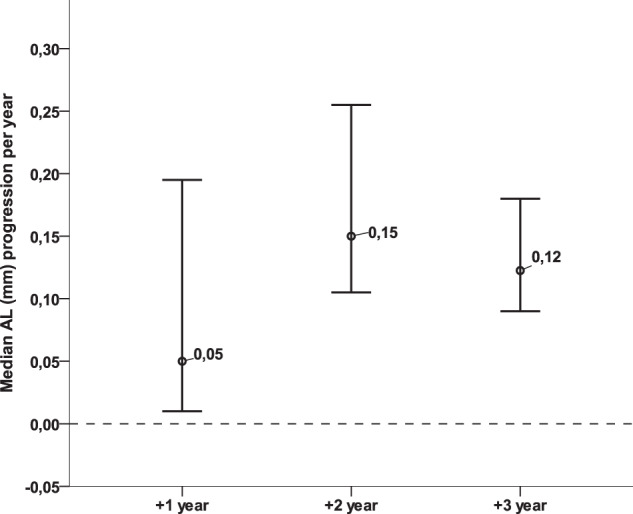
Median Axial Length (AL) change per year in millimeters in children treated with atropine 0.5% for progressive myopia. Error bars represent the 95% Confidence Interval.

The correlation between SER and AL measured during the study was strong with Pearson’s *R*: 0.82 (*p* < 0.01). Annual progression of AL was 0.04 mm in the 1st year, and 0.16 and 0.14 mm in the 2nd and 3rd year, respectively (Fig. 2). We could not compare these progressions with those prior to treatment, as AL had not been measured by the referring clinics 1 year prior to treatment.

**Fig. 3:**
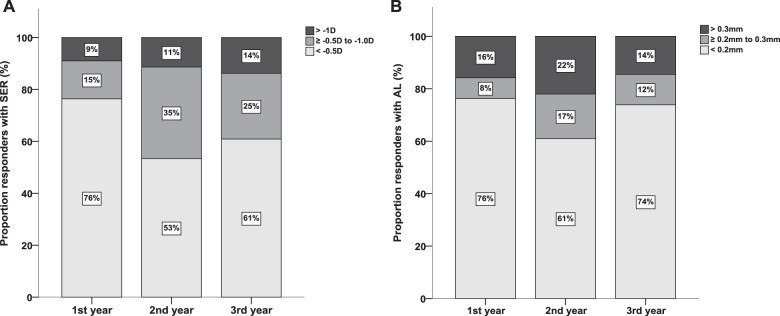
Proportion of good (light gray), moderate (dark gray), and poor (black) responders with respect to spherical equivalent of refraction (**a**) and axial length (**b**) in children on therapy for 3 years.

With respect to treatment response, 76% of children stayed stabilized within −0.5D of SER progression during the 1st year; and 53 and 61% in the 2nd and 3rd year, respectively (Fig. 3a). AL progression in the 1st year stayed within 0.2 mm in 76%; in the 2nd year in 61%, and in the 3rd year in 74% (Fig. 3b).

Age was moderately but significantly related to the treatment effect (Pearson’s *R* for SER: 0.31, *p* < 0.01; for AL: 0.55, *p* < 0.01). Children younger than 10 years of age at the start of therapy had lower treatment effect (median annual progression rate for SER: −0.29D, IQR: 0.44; for AL: 0.20, IQR: 0.18) than older children (median annual progression rate for SER: −0.19D, IQR: 0.41; for AL: 0.06, IQR: 0.08). None of the other determinants at baseline (SER; ethnicity; gender) were significantly associated with annual progression rate during treatment.

We increased the dose of atropine to 1% in 32/89 (36.0%) children (median progression: −0.69D/year, IQR: 0.72; AL: 0.39 mm/year, IQR: 0.19) after a median time of 18 months. This did not diminish progression rates substantially: rates were SER: −0.63D/year (IQR: 0.85) and AL: 0.34 mm/year (IQR: 0.30) during the remaining time of the study.

Aside from the photophobia and reading difficulties, other reported adverse events were nightmares by one child and deterioration of behavioral problems in a child with ADHD. No serious adverse events such as tachycardia, acute angle-closure glaucoma, pyloric obstruction, or asthma were reported.

## Discussion

This study aimed to investigate the effectiveness of atropine for progressive myopia in a European clinical setting. We treated 124 children who presented with either a high degree of myopia or a high progression rate of SER with atropine eye drops at a starting dose of 0.5%, and followed these children for 3 years. Of these, 89 (71.8%) were persistent with therapy during the total duration of the study period. Median SER progression rates declined to 0.00D in the 1st year and to −0.41 and −0.38D in the 2nd and 3rd year, respectively. This corresponded well with a median progression rate for AL of 0.04 mm in the 1st year, and 0.16 and 0.14 mm in the 2nd and 3rd year, respectively. Despite the slightly lower effect in the 2nd and 3rd year, 61% of children still had <−0.5D of SER progression, and 74% had <0.2 mm AL elongation during the last year of the study. After the 1st year, 32/89 of patients progressed 0.3 mm or more while on the starting dose, and were switched to atropine 1%. By contrast, 26/89 stabilized to 0.1 mm/year or less, and were allocated to lower dosages. An important determinant of treatment effect was age: those older than 10 years at baseline remained more stable than those younger.

Given the design of this clinical trial, this study has strengths and limitations. We chose to study high-dose atropine in a real world setting because randomized controlled trials had already demonstrated ample evidence of safety and efficacy of this treatment [10, 15, 20–24]. Our primary intention was to investigate its implementation in Europeans, and our clinical setting enabled great generalizability of findings. Other merits of the study were the long follow-up period and detailed investigation including cycloplegic refraction and AL. A limitation of our design was the use of pretreatment SER progression rates as a reference rather than a separate control group [25]. It is known that myopia progression rates slow down with age, and this effect may have influenced our findings [26]. In all children who prolonged therapy an initial arrest of the myopia progression was seen in the 1st year but median progression continued in the 2nd and 3rd year with −0.41 and −0.38D. However, most progression in those who dropped out of therapy continued at higher rates (−0.9D), implying that treatment effects were real. It is plausible that those whose myopia progressed at a higher rate would be more likely to be referred to our clinic and participate in this study.

Although atropine 0.01% is becoming widely accepted due to minimal side effects and is the preferred treatment in several established practice guidelines, the reported efficacy is lower than that of high-dose atropine [27–29]. The ATOM study showed twice as much control with atropine 0.5 vs. 0.01% (annual progression of SER: −0.24D vs. −0.46D; of AL: 0.19 vs. 0.33 mm) and the LAMP study found a similar dose effect when comparing 0.05 to 0.01% (annual progression of SER: −0.27D vs. −0.59D; of AL: 0.20 vs. 0.41 mm) [15, 30] In our study on children with already high refractive errors (median SER: −5.03D), we aimed to achieve the best possible myopia control. Our data complement the earlier randomized controlled trials in Asians, as atropine 0.5% in our study demonstrated similar responses as ATOMII (Median annual SER: −0.25D; AL: 0.11 mm) [10, 15].

Seventeen children ceased therapy, most in the first months after the start, because of disturbances of accommodation or photosensitivity; 9 children stopped atropine because of an allergy, mostly due to an allergic conjunctivitis; and 2 stopped because of mild non-eye-related reasons. Nine children were lost to follow-up and did not return after their initial start of therapy. Serious systemic adverse events affecting heart, lung, or intestines described for other routes of atropine administration did not occur. Comparing our data to the 0.5% users of the ATOM study, we noticed many similarities [15]. The proportion of reported allergic conjunctivitis was slightly higher (7/124; 5.6%) probably related to the preservative benzalkonium chloride. Our study on mostly European children had more dropouts (*N* = 26; 21%) than studies on the more pigmented Asians (13.7%). Similar to ATOM, we found that photosensitivity complaints were predominantly reported in the first months of treatment; these diminished after 3 months [15, 19]. Adverse events more often led to nonadherence in teenagers than in younger children. Taken together, these observations suggest that remedies addressing the adverse events of high-dose atropine are warranted. We suggest the prescription of photochromic progressive spectacles and a cap for outdoor activity.

This clinical trial shows that findings from the ATOMII trial can be applied to clinical practice, also in Europe. The high-dose atropine group in ATOM I and II experienced strong reduction of the annual myopia progression rate with close to stabilization of SER (+0.03 ± 0.5D) in the 1st year; and mild progression of −0.28 ± 0.92D in the 2nd year [10]. In our study, complete stabilization of SER (0.00D) was achieved during the 1st year. Progression of SER during the 2nd year was −0.41D, albeit somewhat higher than the reduction under trial circumstances. Two other observational studies reported long-term results after high-dose atropine, both were executed in mild myopes > 25 years ago and showed close to stability of refractive error [31, 32]. Our study reports long-term follow-up of more severe myopes on high-dose atropine, and our data shows that progression during the 3rd year (−0.38D) did not increase further, showing stabilization of atropine efficacy. Despite the fact that myopia progression diminishes with age and some of the effect seen during our 3-year follow-up reflects the natural reduction of progression, no significant difference (*p* = 0.08) in progression could be detected between children 10 years or younger, or older children. An intriguing question is whether atropine therapy has a lower effect on myopia progression in Europeans than in Asians. Comparison of annual progression rates shows that atropine 0.5% leads to −0.22D/year in Asian randomized trials and to −0.24D/year in other Asian studies, while atropine 0.5% in our European study leads to a median annual progression of −0.24D/year over a 2-year study period [10, 21, 33]. These figures suggest that ethnic differences in efficacy are minimal.

The biological effect of atropine, a nonselective muscarinic receptor antagonist, remains unclear. The retina and sclera have been suggested as target sites since both tissues harbor muscarinic acetylcholine receptors (mAChR) [34]. A study in guinea pigs found that atropine treatment decreased a regulator of G-protein signaling (a group of mAChRs) mRNA expression and increased collagen type I mRNA expression in sclera. More conclusive evidence whether blockage of mAChR directly interferes with axial elongation is lacking [35]. Several animal studies suggest that atropine therapy prevents eye growth through nitric oxide (NO) production; inhibition of NO interferes with atropine’s effect [36]. Other indirect effects may be through dopamine, as studies have shown that intravitreal injections of atropine cause dopamine release in the retina [37]. Both NO and dopamine are known to act as stop signals for myopia progression [38].

We propose that atropine treatment should be customized according to age, risk of high myopia, and coping capacity with adverse events. One-third of the patients stayed on the starting dose 0.5% atropine, 29% responded so well after 1 year that the dose could be tapered. Lowering the dose did not lead to increased growth, and whether stopping causes a rebound phenomenon remains to be seen as this study continues. One-third responded rather poorly and was switched to the highest dose of atropine. Children who continued on atropine 0.5% or lower dosages showed a median annual progression rate of, respectively, −0.19D (IQR: 0.3) and −0.08D (IQR: 0.3). A stronger efficacy for atropine 1% has been well established by animal research as well as many clinical studies [15, 25, 39]. Children who needed the 1% treatment had an average median annual progression of −0.52D (IQR: 0.4) while on atropine 0.5%, they had a younger median age (*p* < 0.01) and were more myopic at baseline, albeit not significantly (−5.81D (IQR: 3.69) vs. −4.63D (IQR: 3.47), *p* = 0.22). The ATOM study disclosed the same risk factors for poor responders [40]. Unfortunately, switching to atropine 1% in those responding poorly, only slightly diminished growth further in our study. To prevent rebound growth, teenagers who reached stability of AL were tapered in atropine dose before stopping. This strategy prevented rebound of SER and AL, which did occur when high-dose atropine was abruptly stopped in those with allergic reactions. These nine children had an initial good SER response of −0.4D/year (IQR: 0.7) in the 1st year increased to −0.9D/year (IQR: 1.3) in the 2nd year (Table 2).

In summary, this real world study provided SER and AL outcomes for 0.5% starting dose atropine in European children with progressive myopia. We addressed side effects, prescribed photochromic progressive spectacles at the start of the study, and diminished the risk of rebound growth by tapering the dose in children who had a stable SER and AL. With this regimen, 89/124 (71.8%) children stayed on therapy for 3 consecutive years. Median annual progression of SER for children on therapy was −0.25D (AL: 0.11 mm), reflecting a nearly 75% reduction of myopia progression when compared with the rate before treatment. Our data imply that high-dose atropine should be considered a treatment option for severely progressing myopia, even in children with fair skin and blue eyes.

## Summary

### What was known before

- Several controlled trials have indicated that high-dose atropine (0.5–1%) for treatment of progressive myopia is the most effective myopia control measure.
- Although effective, not many specialists in myopia control prescribe high-dose atropine.

### What this study adds

- In a real world setting, 72% of children stayed on therapy for 3 years, despite the side effects.
- Similar to the controlled trials, we found the same effect control over myopia progression, also on the long term.
